# Antioxidant Supplements versus Health Benefits of Brief/Intermittent Exposure to Potentially Toxic Physical or Chemical Agents

**DOI:** 10.3390/cimb43020047

**Published:** 2021-07-10

**Authors:** Rafael Franco, Berta Casanovas, Jordi Camps, Gemma Navarro, Eva Martínez-Pinilla

**Affiliations:** 1 Department of Biochemistry and Molecular Biomedicine, School of Chemistry, University of Barcelona, 08028 Barcelona, Spain; bertacasanovas@gmail.com (B.C.); jcampsji7@alumnes.ub.edu (J.C.); 2 Centro de Investigación Biomédica en Red Enfermedades Neurodegenerativas (CiberNed), Instituto de Salud Carlos III, 28031 Madrid, Spain; g.navarro@ub.edu; 3 Department of Biochemistry and Physiology, Faculty of Pharmacy and Food Science, University of Barcelona, 02028 Barcelona, Spain; 4 Department of Morphology and Cell Biology, Faculty of Medicine, University of Oviedo, 33006 Oviedo, Spain; 5 Instituto de Neurociencias del Principado de Asturias (INEUROPA), 33003 Oviedo, Spain; 6 Instituto de Investigación Sanitaria del Principado de Asturias (ISPA), 33011 Oviedo, Spain

**Keywords:** diet, hormesis, antioxidant, innate mechanisms, oxidative stress, redox, UV radiation

## Abstract

Although antioxidants can act locally to react with an oxidant, oral administration of “antioxidants” is quite useless in treating oxidative stress in tissues. Furthermore, it does not make sense to consider a vitamin as an antioxidant, but vitamin B3 leads to the in vivo formation of compounds that are essential for reducing this stress. A rigorous treatment of the subject indicates that to deal with oxidative stress, the most direct approach is to enhance the innate antioxidant mechanisms. The question is whether this is possible through daily activities. Diets can contain the necessary components for these mechanisms or may induce the expression of the genes involved in them. Another possibility is that pro-oxidant molecules in food increase the sensitivity and power of the detoxification pathways. This option is based on well-known DNA repair mechanisms after exposure to radiation (even from the Sun), or strong evidence of induction of antioxidant capacity after exposure to powerful pro-oxidants such as H_2_O_2_. More experimental work is required to test whether some molecules in food can increase the expression of antioxidant enzymes and/or improve antioxidant mechanisms. Identifying effective molecules to achieve such antioxidant power is critical to the food and nutraceutical industries. The potential of diet-based interventions to combat oxidative stress must be viewed from a new perspective.

## 1. Health and Health Benefits are Human Inventions

Nonhuman animals do not care about the proper functioning of their bodies or about death; they just live and die. Even hominids probably did not care much about health. Today, we human beings want to live as many years as possible and in good shape, so we take pills and supplements to live longer and with little discomfort. We are particularly concerned with chronic diseases, especially those for which there are no pharmacologically effective treatments (e.g., Alzheimer’s and Huntington’s diseases). Consequently, we look for ways to reduce the risk of suffering from any of these pathologies. Thus, there are quite a few interventions that can be beneficial, including, among others, meditation [1,2,3], caffeine consumption [4,5,6,7], and antioxidant supplementation [8,9,10,11]. This perspective article aims to provide an up-to-date view of some of the known or suspected molecules in food that can train our body to better deal with everyday physical or chemical stressors.

## 2. The Multiple Definitions of Antioxidants

According to Encyclopædia Britannica an antioxidant is: “Any of various chemical compounds added to certain foods, natural and synthetic rubbers, gasolines, and other substances to retard autoxidation, the process by which these substances combine with oxygen in the air at room temperature. Retarding autoxidation delays the appearance of such undesirable qualities as rancidity in foods, loss of elasticity in rubbers, and formation of gums in gasolines. Antioxidants most commonly used are such organic compounds as aromatic amines, phenols, and aminophenols”. This definition may not be accurate from a rigorous scientific point of view, but it gives the correct idea of what has been, until recently, considered an antioxidant [12,13,14], that is, a compound that prevents food from spoiling, gasoline from oxidation, etc.

Unfortunately, the word “antioxidant” is now used synonymously with a molecule that provides health benefits, although this is incorrect in many cases. In fact, the term “antioxidant” is now regarded as defined by Wikipedia, which is an unreliable source of information. However, the definition in Wikipedia fits well with the idea that has been transmitted recently, even by scientists: “Antioxidants are molecules that relieve oxidative stress by preventing the formation and oxidation of free radicals [12]. Antioxidants donate one of their electrons or hydrogen to free radicals, stopping their chain reaction [13]. Found in our diet (for example, in vitamins) or formed inside our body (like enzymes), antioxidants can protect us from the damaging effects of free radicals [14]” (refs from Wikipedia). Nevertheless, the information in these sentences is incorrect. On one hand, antioxidants can prevent the formation of free radicals in a test tube, but rarely in vivo [15]. Any compound, antioxidant or not, can donate or acquire electrons. Therefore, an antioxidant can be almost any molecule with few exceptions, e.g., noble gases. On the other hand, a serious misunderstanding consists in considering that a vitamin is an antioxidant. Certainly, a vitamin is necessary for animal/human life, but it is irrelevant whether it is prone to oxidation or prone to reduction, that is, whether the compound is capable of donating or acquiring electrons from other molecules. In fact, neither vitamin K, nor A, nor E, nor any other vitamin should be considered antioxidants [16,17,18]. In a test tube, vitamins can donate or acquire electrons, but they do not act as “antioxidants” in living organisms. Indeed, vitamin C tends to give electrons to other molecules, being an antioxidant, but its function as a vitamin is not related to this property [19]. Excess vitamin C is excreted in the urine in its intact form, that is, it is not oxidized in significant amounts in the human body. Consequently, it is important to define exactly what we are looking for when developing approaches to improve our well-being and reduce the risk of disease. As a suitable example, vitamin B3 (niacin) is required for the synthesis of NAD^+^/NADH and NADP^+^/NADPH, which are key components in metabolism but also in antioxidant detoxification mechanisms [20]. Thus, vitamin B3 is not an antioxidant, but it is key to keeping many physiological processes fully functional, including those related to the inactivation of free radicals or other harmful “oxidant” molecules [21,22]. 

Antioxidant food preservation additives are considered by some to be harmful; there is an increase in organic food choices and a current trend towards preservative-free foods. However, antioxidants are also sought for health benefits [15,23,24]. In short, are antioxidants harmful or beneficial? The answer to this puzzle leads us to the current misconception of what an antioxidant is. Only chemistry has the correct responses.

## 3. The Precise Redox Rules of Chemistry

Life on Earth, except for a few organisms, appeared and evolved in the presence of O_2_. Thus, considerable amounts of O_2_ are needed for cell survival and adaptation to a changing environment and stress, to regulate important metabolic processes and, most importantly, to maintain superior brain functions [25,26]. However, metabolism through redox reactions is invariably associated with the formation of reactive oxygen species (ROS) such as superoxide (O_2_^2−^), hydrogen peroxide (H_2_O_2_), and hydroxyl radical (OH·). When the cellular concentration of ROS is maintained in a physiological range, these species are able to activate signaling pathways that promote biological processes such as cell proliferation or differentiation, which has been termed “oxidative eustress” or “redox biology” [27,28,29,30]. On the contrary, the so-called “oxidative distress” is characterized by excessive levels of ROS that result in damage to DNA, RNA, protein or lipids and consequently in cell injury and death [27,28]. In this sense, the implication of oxidative distress in certain pathologies such as neurodegenerative diseases and cancer has gained momentum in recent decades, and research efforts have focused on the evaluation of specific ROS targets in redox signaling pathways [31]. In mammals, there are various proteins highly sensitive to oxidation with potential functions as redox signalling targets, e.g., the nuclear factor erythroid 2-related factor 2 (NRF2) and the nuclear factor-kB (NF-kB). In fact, NRF2 is a transcription factor that regulates the expression of some genes that encode proteins that participate in detoxification mechanisms [25,32]. In response to oxidative and electrophilic stresses, NRF2 enters into the nucleus and activates antioxidant-specific gene transcription (e.g., NAD(P)H quinone oxidoreductase 1, hæm oxygenase 1, glutamate-cysteine ligase or glutathione S transferases) by “antioxidant response elements” (AREs) present in the promoter region of such target genes [31]. Today, NRF2 in conjunction with Kelch-like ECH-associated protein 1 inhibitor (KEAP1) is known to act as a thiol-based sensor-effector device [32,33]. In default mode, KEAP1 binds to NRF2 and promotes its degradation mediated by ubiquitination, thus preventing the induction of gene expression. However, the conformational alterations of KEAP1, due to the oxidation of some of its cysteine residues, prevent the degradation of NRF2, leading to its nuclear translocation and action upon gene expression [31,34]. Studies in human and animal models of different diseases such as cancer, cardiac pathologies, kidney disorders, diabetes or obesity have revealed a protective effect of the positive regulation of NRF2 in reducing the severity of the disease [35,36]. Interestingly, it has been demonstrated that consuming a diet rich in cruciferous vegetables can affect the KEAP1-NRF2 pathway [37]. In fact, sulforaphane, the main bioactive compound in broccoli sprouts, has been shown to be capable of inducing antioxidant and detoxifying enzymes in a NRF2-dependent manner with proven efficacy in preserving health [38,39,40].

For its part, the transcription factor NF-kB controls many genes involved in inflammatory and immune responses. Despite the complex scenario, what is clear now is that H_2_O_2_ modulates NF-kB activation in two possible ways [41,42]. On the one hand, the oxidation and degradation of the cytoplasmic NF-kB inhibitor, IkB, by H_2_O_2_ may activate NF-kB pathways, i.e., NF-kB enters into the nucleus and activates target genes. On the other hand, the intranuclear redox state may alter expression at the level of a single gene since H_2_O_2_ disrupts NF-kB/DNA binding and blocks transcriptional activity [41,43,44].

Redox processes require both an oxidation half-reaction and a reduction half-reaction. A given compound can act as an electron donor in one redox reaction or as an electron acceptor in another redox reaction. Hydrogen (H) is considered the reference compound and the reducing reaction: 2 H^+^ + 2 e^−^ = H_2_ is characterized by the reduction potential (zero in standard conditions). Therefore, H^+^ can react with electron donors, thus behaving as an oxidant and, in the same way, H_2_ can react with electron acceptors, thus behaving as a reducing agent (antioxidant) [45,46]. In short, depending on the reduction potential, two compounds may or may not react, and a given substance may be oxidized by one compound (oxidant) while it can be reduced by a different compound (antioxidant) [47,48]. Few molecules are only pro-oxidant or only antioxidant. In fact, most compounds can be both pro-oxidants and antioxidants; the only exception is a noble gas that is inert.

A key molecule in mammals is nicotinamide adenine dinucleotide phosphate (NADP), whose oxidized (NADP^+^) and reduced (NADPH) forms coexist, for example, in red blood cells (erythrocytes). As above mentioned, the synthesis of this compound requires vitamin B3. NADPH can donate electrons to an oxidant and become NADP^+^, whereas NADP^+^ can accept electrons from a reductant (antioxidant) and become NADPH. The intake of antioxidants, in the doses in supplements, will not significantly change the ratio of NADP^+^/NADPH in erythrocytes. However, a correct balance of NADP^+^/NADPH is crucial to preserve red blood cells whose intracellular environment is obviously very oxidizing [26]. Red blood cells contain hemoglobin, which is used to transport oxygen and carbon dioxide throughout the body, but its proper function depends on keeping its prosthetic group (Fe^2+^-hæm) in its reduced state in a mechanism involving the NADP^+^/NADPH redox pair. The important thing is to have the innate detox machinery ready to quickly convert NADP^+^ to NADPH. In the case of erythrocytes, this mechanism involves the action of glucose-6-phosphate dehydrogenase (G6PDH) and glutathione, a tripeptide, plus the energy provided by glucose. In summary, erythrocytes depend on glucose supply, sufficient levels of G6PDH, and appropriate glutathione content to survive [49,50]. Therefore, to ensure that an antioxidant strategy is successful, both direct antioxidant mechanisms (through rapid redox reactions) and indirect ones (feeding and stimulation of metabolic detoxification pathways) should be investigated [15,26].

## 4. The Conflicting Methods to Determine Antioxidant Action

As elsewhere described [16], surrogate methods for measuring the antioxidant power of a substance are very often unreliable. A simple example is sucrose, as standard in vitro techniques based on Fehling’s reagent reduction of Cu^2+^ to Cu^+^ are not suitable as they do not measure the reduction potential, which is the only reliable parameter in any redox context. Sucrose does not actually reduce Fehling’s reagent, but it is an in vivo antioxidant. After conversion to fructose and glucose, both sugars are oxidized in human cells. Fehling’s reagent has been instrumental in measuring glucose levels in human blood serum/plasma, but it is useless in evaluating the in vivo antioxidant potential of a molecule.

The supposed antioxidant power of the peptides derived from the hydrolysis of plant albumins is, in our opinion, among the worst cases of misinterpretation of the meaning of “antioxidant”. Stating that peptides are antioxidants is not appropriate if the explanation is not based on considering the only amino acid (in proteins) that is prone to redox reactions in the mammalian body, cysteine. Unfortunately, articles that “demonstrate” the antioxidant properties of rapeseed protein hydrolysates are not the exception [51], but the norm among scientists seeking to demonstrate that many plant compounds are antioxidants or can be converted into antioxidants in vivo. The antioxidant capacity of a compound is given by its reducing potential. There is no parameter that, according to the rules of chemistry, can measure the antioxidant potential of a mixture, a vegetable extract or a hydrolysate. Methods have been developed to help the field to nominate compounds that are a kind of “false positive” antioxidants. One of them, the oxygen radical absorbance capacity (ORAC) test, is presented as reliable and very sophisticated [52]. Upon close inspection of one of the commercially available kits, we found that it consists of measuring the loss of fluorescence of a fluorescent probe when it reacts with an antioxidant. This method is conceptually similar to the one developed by Fehling, that is, it can be used to quantify in vitro the amount of a certain compound in a sample [53,54]. Another commonly used method is the 2,2′-diphenyl-1-picrylhydrazyl (DPPH) free radical scavenging assay. Although with modifications, due to the development of advanced instrumental techniques, the method is quite simple. Pure compounds or extracts are mixed with this stable free radical and the radical scavenging or hydrogen donor potential is measured using a spectrophotometer [55,56,57]. These two are just examples of the variety of assays aimed at assessing antioxidant and radical scavenging activity of foods/beverages that can be found in the literature [58,59,60,61,62]. An absolute “antioxidant” parameter can only be reliably provided by determining the reducing potential and comparing it to the reducing potential of the oxidant being targeted in vivo. Even if the two potentials indicate that detoxification is possible, it would still be a question of whether the reaction can be achieved in seconds, either spontaneously or by the presence of an ad hoc enzyme in the target cell/tissue.

In summary, one can start using the Fehling and ORAC protocols, but to ensure that a compound is an antioxidant would require: (i) in vivo detection of the amount of intact compound and of the metabolites resulting from its oxidation, (ii) measuring the level of compounds necessary for detoxification (e.g., glutathione), and/or (iii) measuring the activity of enzymes involved in detoxification mechanisms (e.g., specific oxidases/reductases/peroxidases). In addition, if the evidence comes from animal models, confirmation in humans is required, as it is mandatory for therapeutic drugs.

## 5. Scientific Evidence in Support of the Need for Boosting Repair Mechanisms

### 5.1. The Classical Case of DNA Repair after Exposure to Harmful Radiation

Alterations in DNA due to Sun exposure mainly consist of thymine dimer formation. About 60 years ago, an enzyme in yeast was observed to lead to the disappearance of the thymine dimer [63]. It should be noted that thymine dimer formation was soon associated with neoplastic transformation [64]. There are different mechanisms of DNA repair after the damaging effects of ultraviolet (UV) radiation or mutagenic agents; DNA photolyase is involved in one of them [65]. Surprisingly, experiments in *Escherichia coli* showed that UV radiation induced the expression of components (enzymes) of DNA repair mechanisms (see [66,67] for early reviews). Certainly, the induction of these components by exposure to agents that alter the structure of DNA has been demonstrated in mammals. The general mechanism related to DNA repair in eukaryotes is also known as “tolerance” since the deleterious agent often first increases the expression of factors that subsequently enhance the transcription of genes encoding detoxification/repair enzymes [68]. 

As seems to have been first pointed out by Philippus Aureolus Theophrastus Bombastus von Hohenheim (Paracelsus): (i) no substance is safe, and (ii) the dose produces the poison, meaning that the dose can turn a molecule generally considered safe into a poison. Taking another perspective, poisons, such as arsenic, were used in low doses to fight disease. The question is whether exposure to the venom can be beneficial. Hormesis is sometimes used to indicate that depending on the dose, exposure to a compound may be beneficial. The word “hormesis” was coined by Southan and Ehrlich [69], who in 1943 reported how the growth of wood-decomposing fungal cultures is affected by different concentrations of heartwood extracts of red cedar (*Thuja plicata*). However, there has been no consensus on how to use the word that has been mistakenly linked to homeopathy [70].

UV light from the sun not only keeps DNA repair mechanisms in good condition, but it is also necessary for the synthesis of vitamins. The solid scientific basis for benefits after sun exposure can be questioned as the line between low dose and toxic exposure is imprecise. Similarly, it can be hypothesized that exposure to food is beneficial, as humans have evolved to cope with both components of food: the good and the bad. Aside from the fact that each molecule can be harmful depending on the dose, food contains thousands of compounds and some of them (in pure form) could be considered harmful/poisonous. We argue that it is irrelevant and imprecise to speak in terms of good/bad UV doses or good/bad food components.

The induction of enzymes that repair DNA not only serves to deal with mutations due to sun exposure, but also to mutagenic compounds or nuclear radiation. Survivors of atomic bombings or nuclear plant catastrophes can be subjected to biomedical studies regardless of the dose, which varies depending on the distance and the material or environmental barriers that attenuate the radiation (see, among others, [71,72,73,74,75,76,77]). Demonstrating benefits due to exposure to radiation is not easy but we have found a genomics study in which gene expression was compared in samples of human blood cells irradiated, ex vivo, under three total doses (0.56, 2.23 and 4.45 Gy) and two different administration regimes for each dose, acute (1.03 Gy/min) or low-dose rate (3.1 mGy/min) [78]. The raw data is available at https://www.ncbi.nlm.nih.gov/geo/query/acc.cgi?acc=GSE65292 (accessed on 31 January 2021). We performed data comparison using the GEO2R tool and selected the 250 genes with lower p values, i.e., with higher confidence, for each of the six conditions (three doses either in acute or low dose exposure). The list of genes for the dose of 0.56 Gy that are common in both lists (250 for acute and 250 for low dose) is given in Table A1 (The lists in Table A1 and Table A2 present the data after curation by eliminating genes with so-called “non-coding sequence” and genes whose products are not well characterized (e.g., SIX homeobox 6 or ribosomal protein S27-like). Data in Table A1 show that radiation exposure is not neutral for the lowest dose (0.56 Gy). Obviously, radiation is neither an antioxidant intervention nor the answer for acquiring health benefits and maintaining well-being as one ages. Interestingly, “antioxidant” enzymes such as ferredoxin reductase are upregulated and glucose-6-phosphate reductase is downregulated with acute but not with low dose administration. A similar comparison for the highest dose (4.45 Gy) (Table A2) detects some genes that are coincident with those in Table A1 but, as expected, the extent of the changes, is higher.

### 5.2. Boosting Repair Mechanisms after Exposure to Foods and Food Supplements

It is a fair assumption that exposure to a pro-oxidant compound, that reinforces innate detoxification mechanisms, may be beneficial against exposure to the same of to another pro-oxidant (reviewed in [15]). In fact, innate mechanisms do not discriminate the nature of the stressor. Expanding the frontier a bit further, boosting innate antioxidant mechanisms serves to cope with re-exposure to the pro-oxidant but also to combat oxidative stress that occurs upon aging or upon intense exercise (oxidative stress is quite remarkable in sedentary aged people) [79,80,81]. In summary, the intake of substances that stimulate antioxidant mechanisms serves to cope with pro-oxidant compounds, including drugs, and to better address with the increase in oxidative stress load in aging.

Examples of data indicating alterations after exposure to foods or chemicals present in foods are here provided. Individuals with congenital G6PDH deficiency have compromised innate detoxification mechanisms (see [82] and references therein); patients cannot consume certain medications (e.g., primaquine) or oxidizing foods, including beans [49,83,84,85]. In fact, fava beans contain harmful natural compounds, vicine and convicine, which cause an overload of oxidative stress in erythrocytes. These cells do not have sufficient G6PDH activity to cope with this situation and, consequently, hemolysis occurs [26]. Therefore, these patients must be careful with some foods and with drugs that challenge the detoxification mechanisms of the human body. Although there is a study in chickens fed diets rich in vicine/convicine, unfortunately, it was not designed to prove health benefits and it cannot be extrapolated to humans [86]. For its study in humans, one would need to measure key redox players in red blood cells (mainly glutathione and G6PDH activity) in volunteers, first under a bean-free diet and, secondly, after fava-bean-containing diets. This research is easy to undertake as it is not a clinical trial; human blood is the most informative source for non-invasive medical-related research.

Rat hepatocytes exposed to some isothiocyanates exhibit an increase in the expression of “antioxidant” enzymes: hæm oxygenase-1 and NAD(P)H:quinone oxidoreductase. Other isothiocyanates enhance the activity of these enzymes by acting at the protein and/or at the transcriptional level [87]. Since similar compounds are found in seasonings, e.g., in mustard [88], it can be assumed that dietary intake of isothiocyanates in food improves the antioxidant capacity of the mammalian liver. Also in rodent liver cells, a study on D-galactose-induced aging reported that an isothiocyanate present in cruciferous vegetables, sulforaphane, reduced biomarkers of liver damage and oxidative stress while increasing glutathione levels and enzyme (glutathione-S-transferase and catalase) activities [89]. In volunteers recruited in a well-controlled feeding intervention, cruciferous vegetables lead to the induction of glutathione S-transferase-A1/2 in plasma [90]. It would be essential to identify the component of the plant that leads to such an apparently beneficial effect.

As a last example, we have selected one that confuses many people, even scientists. Are fruits beneficial for their absolute antioxidant capability? Consistent with the reasons given earlier in this paper and elsewhere [16], the answer is: No. Any food contains both substances prone to reduction and substances prone to oxidation. So the question is whether eating fruit has health benefits, as it is assumed. We have identified a report using rectal biopsies whose results positively correlate frequent fruit consumption with glutathione/glutathione S-transferase activity in rectal mucosa [91].

Finally, the way of approaching the subject in [92] is interesting since it is anticipated that the degree or the benefit may vary from one individual to another. The authors state that “food allergens are innocuous proteins that promote tolerogenic adaptive immune responses in healthy individuals yet in other individuals induce an allergic adaptive immune response”. For one thing, repeated exposure can lead to tolerance, something that has unfortunately not been fully investigated. On the other hand, the processes that determine the first response, whether it is tolerogenic or not tolerogenic, are still unknown [92]. In the case of food or food components, a given action is likely to produce more or less benefits, but not opposite effects; the previously reported effect of cruciferous plants leads to quantitative but not qualitative differences in the induction of glutathione S-transferase-A1/2 depending on the genotype (GSTT1 versus GSTM1) [90].

## 6. Prooxidant Diets with Health Benefits. A Chimera?

Can the pro-oxidant diet boost our antioxidant machinery? There is no answer for this question. The induction of antioxidant proteins by molecules in food may be the result of several mechanisms acquired during evolution, but also of processes similar to those that occur when exposed to UV light or radioactivity. We have not been able to find any article designed to discover whether an oxidant in a diet can enhance the expression of proteins involved in redox detoxification mechanisms. However, we have found strong evidence that oxidants enhance the expression of components of the innate antioxidant defense system.

In the eighties, a study was carried out that recalls DNA repair by exposure to UV light in *Escherichia coli* exposed to hydrogen peroxide (H_2_O_2_). Although this molecule is toxic and can cause the death of the bacteria, it induces an increase in catalase levels. Furthermore, the author claims that “Exposure of *E. coli* to H_2_O_2_ also resulted in the induction of functions under control of the oxyR regulon that enhance the scavenging of active oxygen species, thereby reducing the sensitivity to H_2_O_2_” [93]. In summary, exposure to an oxidant or UV light induces proteins/mechanisms involved in reversing the effect of the harmful agent. Later, a study carried out in the 1990s in a mouse hepatoma cell line showed that the level of mRNA for hæm oxygenase and metallothionein-1 increased with H_2_O_2_ treatment. The same effect was observed when cells were treated with a free radical generator, menadione. In the case of induction of the metallothionein-1 gene, two regulatory elements are involved. One of the conclusions of the authors of this study is that the induction of these genes by ROS is necessary for ROS scavenging [94]. A few years later, it was again shown that exposure to H_2_O_2_ increases both catalase mRNA levels and enzyme activity [95]_._

Interestingly, the authors of an intervention to induce superoxide dismutase, an “antioxidant” enzyme, administered Protandim to volunteers aged 20 to 78 years. Protandim consists of an extract of five different plants that increases the level of this enzyme and also of catalase in the erythrocytes of human blood. The authors conclude that even “modest induction of the catalytic antioxidants SOD and catalase may be a much more effective approach than supplementation with antioxidants (such as vitamins C and E) that can, at best, stoichiometrically scavenge a very small fraction of total oxidant production” [96]. This fits well with our idea that the level of orally administered antioxidants does not result in actual antioxidant actions, while enhancing antioxidant mechanisms/components definitely can.

**Figure 1:**
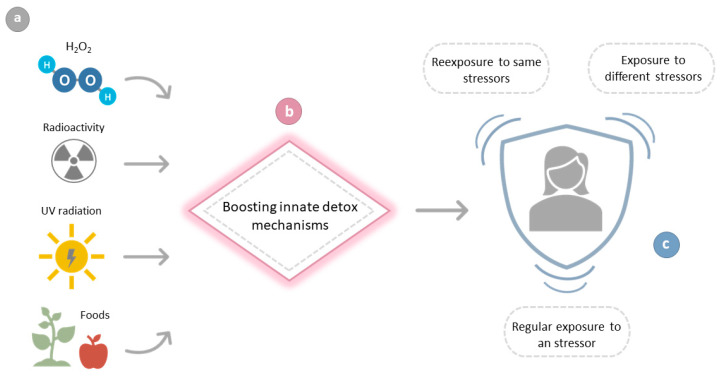
Flow chart illustrating the benefits from a prior exposure to a seemingly noxious agent. Exposure to radioactivity, UV light from the Sun, H_2_O_2_ or some foods or chemicals present in foods (**a**) would increase the levels and/or the activities of antioxidant enzymes, thus improving the innate mechanisms of detoxification (**b**) that cope with the oxidative stress generated by a subsequent re-exposure to the same stressor, by exposure to a different stressor or by the regular exposure to a stressor (**c**).

Noting that exposure to a harmful agent, be it UV light, radioactivity or H_2_O_2_, leads to an enhancement of repair mechanisms; it is tempting to speculate that there are foods that contain pro-oxidants that would induce the expression of components of the innate ROS detoxification mechanisms (Figure 1). A diet that enhances the innate mechanisms that cope with oxidative stress would be considered as “antioxidant.” Accordingly:

Following the example of erythrocyte detoxification mechanisms, the beneficial nature of food components can easily be tested. The experiments, for example, would consist of recruiting healthy volunteers and measuring the glutathione content of red blood cells and the activity of G6PDH after a week of starvation of fava beans and after 1 to 7 days of eating diets containing fava beans.

It would be essential to discover the components of food that lead to increased activity of antioxidant enzymes or glutathione levels, in humans (blood and biopsies) and in animal models of disease (liver, brain, etc.).

We wonder if it is necessary to demonstrate the benefits in all their magnitude, that is, by re-exposure to the stressor. For one thing, the stressor may be a different molecule. On the other hand, the stressor can be consumed regularly and the re-exposure would be beneficial (for instance of fava beans). 

Perhaps a balanced diet would make the redox diet unnecessary, but correct human nutrition is a global problem, and special nutritional requirements for aging are hardly considered (exceptions may occur). Then, by identifying the molecules in foods responsible for the benefits in terms of antioxidation, we argue that a diet consisting of a daily meal that fuels the antioxidant mechanisms would provide health benefits that could be measured by ad hoc research. Hæmolytic crises in G6PDH-deficient patients are caused not only by fava beans, but also by falafel, chickpeas, lima beans, peas, black-eyed peas and lentils [97]. It is tempting to speculate that these foods may serve to keep the innate antioxidant mechanisms in optimal conditions in healthy individuals.

## 7. The Role of the Microbiota

In the past, the relevance of the microbiota has been constantly undermined in biomedical research, yet recent rigorous studies show a significant role of the microbiota in both health and disease. There is a link between the gut and the CNS in which the composition of the microbiota plays a key role, for example in Parkinson’s disease (PD), which is caused by the neurodegeneration of dopaminergic neurons in the substantia nigra. Apart from the fact that dopamine levels are high in the intestine, dopamine receptors are expressed in many types of cells within the structural components of the intestine, and constipation is one of the first symptoms of some PD patients (see [98,99,100] for review), intestinal dysbiosis has an impact on disease etiopathology [101,102,103]. Describing the effects of the microbiota on genesis and progression of PD, or any other neurodegenerative disease, is outside the scope of the present article. However, there is a recent article in which a “unifying” theory is proposed to address the link between microorganisms in the human body and PD [104].

Among the actions mediated by the microbiota that can affect the “redox homeostasis” of the whole body, we would like to highlight: (i) that the molecules in food/beverages can be modified by bacteria in the gastrointestinal tract before reaching the blood and being delivered to the different organs, and (ii) that the microbiota can use glucose and other molecules to produce compounds with antioxidant potential for the host.

## 8. Conclusions

The article highlights the need to understand what an antioxidant is and that oral administration of “antioxidants” has little or no effect. It is also important to convey the fact that any antioxidant requires an oxidant for the redox reaction to take place. The key point is the need to keep the innate detoxification mechanisms in good condition, that is, ready to act to inactivate a harmful oxidant. The obvious way to have enough components of the detoxification mechanisms is to consume the precursors in the diet, but also to improve the responsiveness. There is data, mainly from the field of radiation health effects, showing that exposure to radioactivity (or UV radiation from the Sun) prepares the body for further contact. Similarly, exposure to a pro-oxidant such as H_2_O_2_ prepares the body to then deal with another pro-oxidant. Thus, it is anticipated that the consumption of pro-oxidants in the diet is beneficial not only for future exposure to the same stressor, but also for coping with: (i) excess oxidative stress due to other causes, and (ii) increased “physiological” production of free radicals with aging. The generation of experimental data is urgently needed to confirm or refute the hypothesis. Regarding the redox facet, the field of nutrition research should move on to studying the mechanisms and the way to improve its effectiveness to reduce oxidative stress derived from exogenous factors (for example, drugs), endogenous factors (for example, hypoxic exercise), aging, disease (for example, ischemic stroke), etc.

## Data Availability

The data presented in this study are available on request from the corresponding author. The data are not publicly available due to privacy restrictions.

## Declaration

The Molecular Neurobiology laboratory of the University of Barcelona is considered of excellence by the Regional Catalonian Government (grup consolidat #2017 SGR 1497), which does neither provide specific funding for personnel, equipment and reagents nor for payment of services.

## Appendix A

**Table A1:** List of genes for the dose of 0.56 Gy that are common in both lists of the two administration regimes for this dose, acute or low dose.

Gene Symbol	Gene Title	Acute logFC	Low logFC
*AEN*	Apoptosis-enhancing nuclease	3.321	3.210
*TRIOBP*	TRIO and F-actin binding protein	2.793	1.197
*FDXR*	Ferredoxin reductase	2.774	2.637
*DDB2*	Damage-specific DNA binding protein 2, 48kDa	2.071	1.901
*PAPPA*	Pregnancy-associated plasma protein A, Pappalysin 1	1.882	2.502
*CD70*	CD70 molecule	1.827	1.646
*BAX*	BCL2-associated X protein	1.698	1.592
*VWCE*	Von Willebrand factor C and EGF domains	1.660	1.528
*E2F7*	E2F transcription factor 7	1.596	1.364
*ACTA2*	Actin, alpha 2, smooth muscle, aorta	1.531	1.293
*RPS27L*	Ribosomal protein S27-like	1.527	1.329
*TNFSF4*	Tumor necrosis factor (ligand) superfamily, member 4	1.524	1.508
*CCNG1*	Cyclin G1	1.463	1.304
*TNFRSF10B*	Tumor necrosis factor receptor superfamily, member 10b	1.455	1.381
*CDKN1A*	Cyclin-dependent kinase inhibitor 1A (p21, Cip1)	1.197	1.066
*BBC3*	BCL2 binding component 3	1.193	0.923
*OR52N4*	Olfactory receptor, family 52, subfamily N, member 4	1.184	1.369
*GLS2*	Glutaminase 2 (liver, mitochondrial)	1.172	1.117
*RBP4*	Retinol binding protein 4, plasma	1.169	1.226
*POLH*	Polymerase (DNA directed), eta	1.137	0.821
*TNFSF9*	Tumor necrosis factor (ligand) superfamily, member 9	1.131	0.656
*PHPT1*	Phosphohistidine phosphatase 1	1.123	0.847
*KLLN*	Killin, p53-regulated DNA replication inhibitor	0.993	0.902
*XPC*	Xeroderma pigmentosum, complementation group C	0.966	0.957
*SESN1*	Sestrin 1	0.959	0.795
*TNFSF8*	Tumor necrosis factor (ligand) superfamily, member 8	0.925	1.063
*CST2*	Cystatin SA	0.924	1.067
*GDF15*	Growth differentiation factor 15	0.832	1.153
*ACER2*	Alkaline ceramidase 2	0.742	0.966
*KCNN4*	Potassium intermediate/small conductance calcium-activated channel, subfamily N, member 4	0.710	0.688
*PPMD1*	Protein phosphatase, Mg2^+^/Mn2^+^ dependent, 1D	0.692	0.707
*LIG1*	Ligase I, DNA, ATP-dependent	0.631	0.567
*TFAP4*	Transcription factor AP-4 (activating enhancer binding protein 4)	−0.694	−1.163
*C10ORF116*	Chromosome 10 open reading frame 116	−0.791	−0.827
*TAAR8*	Trace amine associated receptor 8	−0.807	−0.740
*KRTAP13-2*	Keratin associated protein 13-2	−0.843	−0.641
*LMX1B*	LIM homeobox transcription factor 1, beta	−0.964	−0.909
*DOK5*	Docking protein 5	−0.976	−1.534
*NR1I3*	Nuclear receptor subfamily 1, group I, member 3	−1.243	−0.890
*ANKRD34B*	Ankyrin repeat domain 34B	−1.248	−1.538
*SCT*	Secretin	−1.318	−1.311
*LMOD2*	Leiomodin 2 (cardiac)	−1.639	−1.845
*RXFP2*	Relaxin/insulin-like family peptide receptor 2	−2.191	−1.529

FC: Fold Change.

**Table A2:** List of genes for the dose of 4.45 Gy that are common in both lists of the two administration regimes for this dose, acute or low dose.

Gene Symbol	Gene Title	Acute logFC	Low logFC
*APOBEC3H*	Apolipoprotein B mRNA editing enzyme. catalytic polypeptide-like 3H	4.746	3.957
*VWCE*	Von Willebrand factor C and EGF domains	4.577	3.579
*FDXR*	Ferredoxin reductase	4.505	3.961
*E2F7*	E2F transcription factor 7	4.108	3.581
*AEN*	Apoptosis enhancing nuclease	4.001	4.155
*TNFSF4*	Tumor necrosis factor (ligand) superfamily. member 4	3.701	2.914
*PAPPA*	Pregnancy-associated plasma protein A. pappalysin 1	3.602	3.379
*PHLDA3*	Pleckstrin homology-like domain. family A. member 3	3.314	2.830
*NTN1*	Netrin 1	3.204	2.227
*DDB2*	Damage-specific DNA binding protein 2. 48kDa	3.025	2.900
*CD70*	CD70 molecule	2.978	2.895
*PCNA*	Proliferating cell nuclear antigen	2.895	2.787
*GDF15*	Growth differentiation factor 15	2.872	2.751
*TMPRSS7*	Transmembrane protease. serine 7	2.777	2.194
*CDKN1A*	Cyclin-dependent kinase inhibitor 1A (p21. Cip1)	2.619	2.297
*ACTA2*	Actin. alpha 2. smooth muscle. aorta	2.400	1.548
*CCNG1*	Cyclin G1	2.390	1.865
*TAC3*	Tachykinin 3	2.367	1.459
*BBC3*	BCL2 binding component 3	2.133	1.867
*POLH*	Polymerase (DNA directed). eta	2.101	1.632
*BAX*	BCL2-associated X protein	2.074	2.134
*GRIN3B*	Glutamate receptor. ionotropic. N-methyl-D-aspartate 3B	2.069	1.856
*GNAT2*	Guanine nucleotide binding protein (G protein). alpha transducing activity polypeptide 2	2.056	2.409
*PHPT1*	Phosphohistidine phosphatase 1	2.037	1.731
*TNFSF9*	Tumor necrosis factor (ligand) superfamily. member 9	2.028	2.195
*FCAMR*	Fc receptor. IgA. IgM. high affinity	1.842	1.704
*LAMC3*	Laminin. gamma 3	1.774	1.444
*SESN1*	Sestrin 1	1.751	1.538
*GNG4*	Guanine nucleotide binding protein (G protein). gamma 4	1.728	1.802
*PIGR*	Polymeric immunoglobulin receptor	1.639	2.238
*ACER2*	Alkaline ceramidase 2	1.628	1.424
*TNFRSF10B*	Tumor necrosis factor receptor superfamily. member 10b	1.599	1.905
*GPR172B*	G protein-coupled receptor 172B	1.574	0.966
*PPM1D*	Protein phosphatase. Mg2^+^/Mn2^+^ dependent. 1D	1.566	1.310
*TNFSF8*	Tumor necrosis factor (ligand) superfamily. member 8	1.559	1.449
*TYMS*	Thymidylate synthetase	1.491	1.579
*LMNA*	Lamin A/C	1.438	1.780
*IER5*	Immediate early response 5	1.417	1.237
*OR52N4*	Olfactory receptor. family 52. subfamily N. member 4	1.388	1.965
*MGAT3*	Mannosyl (beta-1.4-)-glycoprotein beta-1.4-N-acetylglucosaminyltransferase	1.358	0.998
*DHDH*	Dihydrodiol dehydrogenase (dimeric)	1.291	1.124
*LIG1*	Ligase I. DNA. ATP-dependent	1.235	0.885
*APOBEC3C*	Apolipoprotein B mRNA editing enzyme. catalytic polypeptide-like 3C	1.163	1.144
*KCNN4*	Potassium intermediate/small conductance calcium-activated channel. subfamily N. member 4	1.148	0.913
*DRAM1*	DNA-damage regulated autophagy modulator 1	1.141	0.973
*MERTK*	C-mer proto-oncogene tyrosine kinase	1.126	1.101
*MAP4K4*	Mitogen-activated protein kinase kinase kinase kinase 4	1.052	1.003
*ARHGEF3*	Rho guanine nucleotide exchange factor (GEF) 3	1.052	1.090
*F5*	Coagulation factor V (proaccelerin. labile factor)	1.049	1.150
*RGL1*	Ral guanine nucleotide dissociation stimulator-like 1	1.027	1.078
*PTP4A1*	Protein tyrosine phosphatase type IVA. member 1	1.006	0.883
*PRKAB1*	Protein kinase. AMP-activated. beta 1 non-catalytic subunit	1.002	0.872
*ABLIM2*	Actin binding LIM protein family. member 2	−0.929	−0.967
*PPP2R1B*	Protein phosphatase 2. regulatory subunit A. beta	−1.091	−0.900
*OSBPL10*	Oxysterol binding protein-like 10	−1.225	−0.798
*TFAP4*	Transcription factor AP-4 (activating enhancer binding protein 4)	−1.309	−1.188
*CORO2B*	Coronin. actin binding protein. 2B	−1.712	−1.996
*FCRL2*	Fc receptor-like 2	−1.925	−1.498
*CD160*	CD160 molecule	−2.519	−1.765

FC: Fold Change.

## References

[1] Horowitz S. (2010). Health Benefits of Meditation: What the Newest Research Shows. Altern. Complement. Ther..

[2] Herzog H., Lele V.R., Kuwert T., Langen K.J., Rota Kops E., Feinendegen L.E. (1990). Changed pattern of regional glucose metabolism during yoga meditative relaxation. Neuropsychobiology.

[3] Chu L.-C. (2010). The benefits of meditation vis-à-vis emotional intelligence, perceived stress and negative mental health. Stress Health.

[4] Oñatibia-Astibia A., Franco R., Martínez-Pinilla E. (2017). Health benefits of methylxanthines in neurodegenerative diseases. Mol. Nutr. Food Res..

[5] Oñatibia-Astibia A., Martínez-Pinilla E., Franco R. (2016). The potential of methylxanthine-based therapies in pediatric respiratory tract diseases. Respir. Med..

[6] Franco R., Oñatibia-Astibia A., Martínez-Pinilla E. (2013). Health benefits of methylxanthines in cacao and chocolate. Nutrients.

[7] Franco R. (2009). Café y salud mental. Aten. Primaria.

[8] Pham-Huy L.A., He H., Pham-Huy C. (2008). Free radicals, antioxidants in disease and health. Int. J. Biomed. Sci..

[9] Brambilla D., Mancuso C., Scuderi M.R., Bosco P., Cantarella G., Lempereur L., Di Benedetto G., Pezzino S., Bernardini R. (2008). The role of antioxidant supplement in immune system, neoplastic, and neurodegenerative disorders: A point of view for an assessment of the risk/benefit profile. Nutr. J..

[10] Bjelakovic G., Nikolova D., Gluud L.L., Simonetti R.G., Gluud C. (2008). Antioxidant supplements for prevention of mortality in healthy participants and patients with various diseases. Cochrane Database Syst. Rev..

[11] Bjelakovic G., Nikolova D., Simonetti R.G., Gluud C. (2008). Systematic review: Primary and secondary prevention of gastrointestinal cancers with antioxidant supplements. Aliment. Pharmacol. Ther..

[12] Halliwell B., Aeschbach R., Löliger J., Aruoma O.I. (1995). The characterization of antioxidants. Food Chem. Toxicol..

[13] Kaur C., Kapoor H.C. (2008). Antioxidants in fruits and vegetables—The millennium’s health. Int. J. Food Sci. Technol..

[14] Afzal M., Armstrong D. (2002). Fractionation of herbal medicine for identifying antioxidant activity. Methods Mol. Biol..

[15] Franco R., Navarro G., Martínez-Pinilla E. (2019). Hormetic and Mitochondria-Related Mechanisms of Antioxidant Action of Phytochemicals. Antioxidants.

[16] Franco R., Martínez-Pinilla E. (2017). Chemical rules on the assessment of antioxidant potential in food and food additives aimed at reducing oxidative stress and neurodegeneration. Food Chem..

[17] Niki E., Traber M.G. (2012). A history of vitamin E. Ann. Nutr. Metab..

[18] Borel P., Desmarchelier C. (2016). Genetic variations involved in vitamin E status. Int. J. Mol. Sci..

[19] Franco R., Navarro G., Martínez-Pinilla E. (2019). Antioxidants versus food antioxidant additives and food preservatives. Antioxidants.

[20] Kirkland J.B., Meyer-Ficca M.L. (2018). Niacin. Adv. Food. Nutr. Res..

[21] Ying W. (2007). NAD+ and NADH in brain functions, brain diseases and brain aging. Front. Biosci..

[22] Yang Y., Sauve A.A. (2016). NAD+ metabolism: Bioenergetics, signaling and manipulation for therapy. Biochim. Biophys. Acta Proteins Proteom..

[23] Beya M.M., Netzel M.E., Sultanbawa Y., Smyth H., Hoffman L.C. (2021). Plant-based phenolic molecules as natural preservatives in comminuted meats: A review. Antioxidants.

[24] Zehiroglu C., Ozturk Sarikaya S.B. (2019). The importance of antioxidants and place in today’s scientific and technological studies. J. Food Sci. Technol..

[25] Scandalios J.G. (2005). Oxidative stress: Molecular perception and transduction of signals triggering antioxidant gene defenses. Braz. J. Med. Biol. Res..

[26] Franco R., Navarro G., Martínez-Pinilla E. (2019). Antioxidant Defense Mechanisms in Erythrocytes and in the Central Nervous System. Antioxidants.

[27] Sies H., Berndt C., Jones D.P. (2017). Oxidative stress. Annu. Rev. Biochem..

[28] Sies H. (2017). Hydrogen peroxide as a central redox signaling molecule in physiological oxidative stress: Oxidative eustress. Redox Biol..

[29] Schieber M., Chandel N.S. (2014). ROS function in redox signaling and oxidative stress. Curr. Biol..

[30] Zhang L., Wang X., Cueto R., Effi C., Zhang Y., Tan H., Qin X., Ji Y., Yang X., Wang H. (2019). Biochemical basis and metabolic interplay of redox regulation. Redox Biol..

[31] Sies H., Jones D.P. (2020). Reactive oxygen species (ROS) as pleiotropic physiological signalling agents. Nat. Rev. Mol. Cell Biol..

[32] Itoh K., Chiba T., Takahashi S., Ishii T., Igarashi K., Katoh Y., Oyake T., Hayashi N., Satoh K., Hatayama I. (1997). An Nrf2/small Maf heterodimer mediates the induction of phase II detoxifying enzyme genes through antioxidant response elements. Biochem. Biophys. Res. Commun..

[33] Yamamoto M., Kensler T.W., Motohashi H. (2018). The KEAP1-NRF2 system: A thiol-based sensor-effector apparatus for maintaining redox homeostasis. Physiol. Rev..

[34] Kansanen E., Kuosmanen S.M., Leinonen H., Levonenn A.L. (2013). The Keap1-Nrf2 pathway: Mechanisms of activation and dysregulation in cancer. Redox Biol..

[35] Liebman S.E., Le T.H. (2021). Eat your broccoli: Oxidative stress, nrf2, and sulforaphane in chronic kidney disease. Nutrients.

[36] McWalter G.K., Higgins L.G., McLellan L.I., Henderson C.J., Song L., Thornalley P.J., Itoh K., Yamamoto M., Hayes J.D. (2004). Transcription factor Nrf2 is essential for induction of NAD(P)H:quinone oxidoreductase 1, glutathione S-transferases, and glutamate cysteine ligase by broccoli seeds and isothiocyanates. J. Nutr..

[37] Zhang Y., Talalay P., Cho C.G., Posner G.H. (1992). A major inducer of anticarcinogenic protective enzymes from broccoli: Isolation and elucidation of structure. Proc. Natl. Acad. Sci. USA.

[38] Houghton C.A., Fassett R.G., Coombes J.S. (2016). Sulforaphane and Other Nutrigenomic Nrf2 Activators: Can the Clinician’s Expectation Be Matched by the Reality?. Oxid. Med. Cell. Longev..

[39] Dinkova-Kostova A.T., Fahey J.W., Kostov R.V., Kensler T.W. (2017). KEAP1 and done? Targeting the NRF2 pathway with sulforaphane. Trends Food Sci. Technol..

[40] Eggler A.L., Savinov S.N. (2013). Chemical and Biological Mechanisms of Phytochemical Activation of NRF2 and Importance in Disease Prevention. 50 Years of Phytochemistry Research.

[41] Cintra E., Silva D.D.O., Estevanato L.L.C., Simioni A.R., De Andrade Rodrigues M.M., Lacava B.M., Lacava Z.G.M., Tedesco A.C., Morais P.C., Báo S.N. (2012). Successful strategy for targeting the central nervous system using magnetic albumin nanospheres. J. Biomed. Nanotechnol..

[42] Schreck R., Rieber P., Baeuerle P.A. (1991). Reactive oxygen intermediates as apparently widely used messengers in the activation of the NF-κB transcription factor and HIV-1. EMBO J..

[43] Marinho H.S., Real C., Cyrne L., Soares H., Antunes F. (2014). Hydrogen peroxide sensing, signaling and regulation of transcription factors. Redox Biol..

[44] Halvey P.J., Hansen J.M., Johnson J.M., Go Y.M., Samali A., Jones D.P. (2007). Selective oxidative stress in cell nuclei by nuclear-targeted D-amino acid oxidase. Antioxid. Redox Signal..

[45] Herrmann J.M., Dick T.P. (2012). Redox Biology on the rise. Biol. Chem..

[46] Naidu A.S. (2013). Redox Life.

[47] Gitler C., Danon A., Gitler C., Danon A. (2003). Cellular Implications of Redox Signaling.

[48] Sies H. (2015). Oxidative stress: A concept in redox biology and medicine. Redox Biol..

[49] van Zwieten R., Verhoeven A.J., Roos D. (2014). Inborn defects in the antioxidant systems of human red blood cells. Free Radic. Biol. Med..

[50] Ho H., Cheng M., Chiu D.T. (2007). Glucose-6-phosphate dehydrogenase—From oxidative stress to cellular functions and degenerative diseases. Redox Rep..

[51] Pan M., Jiang T.S., Pan J.L. (2011). Antioxidant Activities of Rapeseed Protein Hydrolysates. Food Bioprocess. Technol..

[52] Cao G., Alessio H.M., Cutler R.G. (1993). Oxygen-radical absorbance capacity assay for antioxidants. Free Radic. Biol. Med..

[53] Ou B., Huang D., Hampsch-Woodill M., Flanagan J.A., Deemer E.K. (2002). Analysis of antioxidant activities of common vegetables employing oxygen radical absorbance capacity (ORAC) and ferric reducing antioxidant power (FRAP) assays: A comparative study. J. Agric. Food Chem..

[54] Dávalos A., Gómez-Cordovés C., Bartolomé B. (2004). Extending Applicability of the Oxygen Radical Absorbance Capacity (ORAC-Fluorescein) Assay. J. Agric. Food Chem..

[55] Blois M. (1958). Antioxidant determinations by the use of a stable free. Nature.

[56] Kedare S.B., Singh R.P. (2011). Genesis and development of DPPH method of antioxidant assay. J. Food Sci. Technol..

[57] Sherma J. (2018). Review of the determination of the antioxidant activity of foods, food ingredients, and dietary supplements by thin layer chromatography-direct bioautography, spectrometry, and the dot-blot procedure. J. AOAC Int..

[58] Pohl F., Lin P.K.T. (2018). The potential use of plant natural products and plant extracts with antioxidant properties for the prevention/treatment of neurodegenerative diseases: In vitro, in vivo and clinical trials. Molecules.

[59] Cruciani S., Trenta M., Rassu G., Garroni G., Petretto G.L., Ventura C., Maioli M., Pintore G. (2021). Identifying a role of red and white wine extracts in counteracting skin aging: Effects of antioxidants on fibroblast behavior. Antioxidants.

[60] Vallverdú-Queralt A., Boix N., Piqué E., Gómez-Catalan J., Medina-Remon A., Sasot G., Mercader-Martí M., Llobet J.M., Lamuela-Raventos R.M. (2015). Identification of phenolic compounds in red wine extract samples and zebrafish embryos by HPLC-ESI-LTQ-Orbitrap-MS. Food Chem..

[61] Tai A., Sawano T., Yazama F., Ito H. (2011). Evaluation of antioxidant activity of vanillin by using multiple antioxidant assays. Biochim. Biophys. Acta Gen. Subj..

[62] Martinez R.M., Pinho-Ribeiro F.A., Steffen V.S., Silva T.C.C., Caviglione C.V., Bottura C., Fonseca M.J.V., Vicentini F.T.M.C., Vignoli J.A., Baracat M.M. (2016). Topical formulation containing naringenin: Efficacy against ultraviolet B irradiation-induced skin inflammation and oxidative stress in mice. PLoS ONE.

[63] Wulff D.L., Rupert C.S. (1962). Disappearance of thymine photodimer in ultraviolet irradiated DNA upon treatment with a photoreactivating enzyme from Baker’s yeast. Biochem. Biophys. Res. Commun..

[64] Hart R.W., Setlow R.B. (1975). Direct evidence that pyrimidine dimers in DNA result in neoplastic transformation. Basic Life Sci..

[65] Sancar A., Sancar G.B. (1984). Escherichia coli DNA photolyase is a flavoprotein. J. Mol. Biol..

[66] Radman M. (1974). Phenomenology of an inducible mutagenic DNA repair pathway in Escherichia coli: SOS repair hypothesis. Basic. Life. Sci..

[67] Witkin E.M. (1976). Ultraviolet mutagenesis and inducible DNA repair in Escherichia coli. Bacteriol. Rev..

[68] Hanawalt P.C., Cooper P.K., Ganesan A.K., Smith C.A. (1979). DNA Repair in Bacteria and Mammalian Cells. Annu. Rev. Biochem..

[69] Southan C., Ehrlich J. (1943). Effects of extract of western red-cedar heartwood on certain wood-decaying fungi in culture. Phytopathology.

[70] Boxenbaum H., Neafsey P.J., Fournier D.J. (1988). Hormesis, gompertz functions, and risk assessment. Drug Metab. Rev..

[71] Sutou S. (2019). Black rain in Hiroshima: A critique to the Life Span Study of A-bomb survivors, basis of the linear no-threshold model. Genes Environ..

[72] Jargin S.V. (2018). Hormesis and radiation safety norms: Comments for an update. Hum. Exp. Toxicol..

[73] Kudryasheva N.S., Kovel E.S. (2019). Monitoring of low-intensity exposures via luminescent bioassays of different complexity: Cells, enzyme reactions, and fluorescent proteins. Int. J. Mol. Sci..

[74] Cuttler J.M. (2020). Application of Low Doses of Ionizing Radiation in Medical Therapies. Dose-Response.

[75] Jargin S.V. (2020). Radiation Safety and Hormesis. Front. Public Health.

[76] Fritz-Niggli H. (1995). 100 years of radiobiology: Implications for biomedicine and future perspectives. Experientia.

[77] Hattori S. (1994). Current status and perspectives of research on radiation hormesis in Japan. Chin. Med. J..

[78] Ghandhi S.A., Smilenov L.B., Elliston C.D., Chowdhury M., Amundson S.A. (2015). Radiation dose-rate effects on gene expression for human biodosimetry Functional and structural genomics. BMC Med. Genom..

[79] Morifuji M., Sakai K., Sanbongi C., Sugiura K. (2005). Dietary whey protein downregulates fatty acid synthesis in the liver, but upregulates it in skeletal muscle of exercise-trained rats. Nutrition.

[80] Jamurtas A.Z., Fatouros I.G., Koukosias N., Manthou E., Tofas T., Yfanti C., Nikolaidis M.G., Koutedakis Y. (2006). Effect of exercise on oxidative stress in individuals with glucose-6-phosphate dehydrogenase deficiency. In Vivo.

[81] Georgakouli K., Fatouros I.G., Draganidis D., Papanikolaou K., Tsimeas P., Deli C.K., Jamurtas A.Z. (2019). Exercise in glucose-6-phosphate dehydrogenase deficiency: Harmful or harmless? A narrative review. Oxid. Med. Cell. Longev..

[82] Sodeinde O. (1992). Glucose-6-phosphate dehydrogenase deficiency. Baillieres. Clin. Haematol..

[83] Baker M.A., Bosia A., Pescarmona G., Turrini F., Arese P. (1984). Mechanism of Action of Divicine in a Cell-free System and in Glucose-6-phosphate Dehydrogenase-deficient Red Cells. Toxicol. Pathol..

[84] Vural N., Sardas S. (1984). Biological activities of broad bean (*Vicia faba* L.) extracts cultivated in South Anatolia in favism sensitive subjects. Toxicology.

[85] Luzzatto L., Nannelli C., Notaro R. (2016). Glucose-6-Phosphate Dehydrogenase Deficiency. Hematol. Oncol. Clin. N. Am..

[86] Lessire M., Gallo V., Prato M., Akide-Ndunge O., Mandili G., Marget P., Arese P., Duc G. (2017). Effects of faba beans with different concentrations of vicine and convicine on egg production, egg quality and red blood cells in laying hens. Animal.

[87] La Marca M., Beffy P., Della Croce C., Gervasi P.G., Iori R., Puccinelli E., Longo V. (2012). Structural influence of isothiocyanates on expression of cytochrome P450, phase II enzymes, and activation of Nrf2 in primary rat hepatocytes. Food Chem. Toxicol..

[88] Adegbeye M.J., Reddy P.R.K., Chilaka C.A., Balogun O.B., Elghandour M.M.M.Y., Rivas-Caceres R.R., Salem A.Z.M. (2020). Mycotoxin toxicity and residue in animal products: Prevalence, consumer exposure and reduction strategies—A review. Toxicon.

[89] Saleh D.O., Mansour D.F., Hashad I.M., Bakeer R.M. (2019). Effects of sulforaphane on D-galactose-induced liver aging in rats: Role of keap-1/nrf-2 pathway. Eur. J. Pharmacol..

[90] Navarro S.L., Chang J.L., Peterson S., Chen C., King I.B., Schwarz Y., Li S.S., Li L., Potter J.D., Lampe J.W. (2009). Modulation of human serum glutathione S-transferase A1/2 concentration by cruciferous vegetables in a controlled feeding study is influenced by GSTM1 and GSTT1 genotypes. Cancer Epidemiol. Biomark. Prev..

[91] Wark P.A., Grubben M.J.A.L., Peters W.H.M., Nagengast F.M., Kampman E., Kok F.J., van’t Veer P. (2004). Habitual consumption of fruits and vegetables: Associations with human rectal glutathione S-transferase. Carcinogenesis.

[92] Tomar S., Hogan S.P. (2020). Recent advances in mechanisms of food allergy and anaphylaxis. F1000Research.

[93] Imlay J.A., Linn S. (1987). Mutagenesis and stress responses induced in Escherichia coli by hydrogen peroxide. J. Bacteriol..

[94] Dalton T., Palmiter R.D., Andrews G.K. (1994). Transcriptional induction of the mouse metallothionein-I gene in hydrogen peroxide-treated hepa cells involves a composite major late transcription factor/antioxidant response element and metal response promoter elements. Nucleic Acids Res..

[95] Röhrdanz E., Kahl R. (1998). Alterations of antioxidant enzyme expression in response to hydrogen peroxide. Free Radic. Biol. Med..

[96] Nelson S.K., Bose S.K., Grunwald G.K., Myhill P., McCord J.M. (2006). The induction of human superoxide dismutase and catalase in vivo: A fundamentally new approach to antioxidant therapy. Free Radic. Biol. Med..

[97] Hagag A.A., Badraia I.M., Elfarargy M.S., Abd Elmageed M.M., Abo-Ali E.A. (2018). Study of Glucose-6-Phosphate Dehydrogenase Deficiency: 5 Years Retrospective Egyptian Study. Endocr. Metab. Immune Disord. Drug Targets.

[98] Jost W.H., Schimrigk K. (1991). Constipation in Parkinson’s disease. Klin. Wochenschr..

[99] Mukherjee A., Biswas A., Das S.K. (2016). Gut dysfunction in Parkinson’s disease. World J. Gastroenterol..

[100] Skjærbæk C., Knudsen K., Horsager J., Borghammer P. (2021). Gastrointestinal Dysfunction in Parkinson’s Disease. J. Clin. Med..

[101] Parashar A., Udayabanu M. (2017). Gut microbiota: Implications in Parkinson’s disease. Park. Relat. Disord..

[102] Mulak A., Bonaz B. (2015). Brain-gut-microbiota axis in Parkinson’s disease. World J. Gastroenterol..

[103] Huang Y., Liao J., Liu X., Zhong Y., Cai X., Long L. (2021). Review: The Role of Intestinal Dysbiosis in Parkinson’s Disease. Front. Cell. Infect. Microbiol..

[104] Munoz-Pinto M.F., Empadinhas N., Cardoso S.M. (2021). The neuromicrobiology of Parkinson’s disease: A unifying theory. Ageing Res. Rev..

